# Taxonomic notes and distribution extension of Durga Das’s leaf-nosed bat *Hipposideros durgadasi* Khajuria, 1970 (Chiroptera: Hipposideridae) from south India

**DOI:** 10.3897/BDJ.2.e4127

**Published:** 2014-11-20

**Authors:** Harpreet Kaur, Srinivasulu Chelmala, Bhargavi Srinivasulu, Tariq Ahmed Shah, Gundena Devender, Aditya Srinivasulu

**Affiliations:** † Wildlife Biology and Taxonomy Lab, Department of Zoology, University College of Science, Osmania University, Hyderabad, India; ‡ Natural History Museum and Wildlife Biology & Taxonomy Lab, Dept. of Zoology, University College of Science, Osmania University, Hyderabad, India; § Systematics, Ecology & Conservation Laboratory, Zoo Outreach Organization (ZOO), 96 Kumudham Nagar, Vilankurichi Road, Coimbatore, India; | Biodiversity Research and Conservation Society, 303 Orchid, Kanajiguda, Tirumalgiri, Secunderabad, India

**Keywords:** *Hipposideros durgadasi*, Kolar, India, Hanumanhalli, Therahalli, range extension, morphological description

## Abstract

Durga Das’s leaf-nosed bat *Hipposideros durgadasi* Khajuria, 1970 is endemic to India, and was known only from Katanga, Katangi, and Richhai villages, in Jabalpur district, Madhya Pradesh. During surveys conducted in Kolar district, Karnataka, India, we successfully mist-netted a few individuals belonging to the *bicolor* species group which, upon detailed external, craniodental and bacular studies were identified as Durga Das’s leaf-nosed bat. This paper reports the presence of this species in southern India, extending its distribution range by almost 1300 km. We also provide a detailed morphological description for this species.

## Introduction

The genus *Hipposideros* Gray, 1831 is represented by thirteen species belonging to five species groups in South Asia (Srinivasulu and Srinivasulu 2012). Two species, Durga Das’s leaf-nosed bat (*Hipposideros durgadasi* Khajuria, 1970) and Kolar leaf-nosed bat (*Hipposideros hypophyllus* Kock and Bhat, 1994), belong to the *bicolor* species group and are endemic to India, with records only from Jabalpur district, Madhya Pradesh (Bates and Harrison 1997, Corbet and Hill 1992, Khajuria 1970, Khajuria 1980, Simmons 2005, Srinivasulu and Srinivasulu 2012, Topal 1975) and Kolar district, Karnataka (Bates and Harrison 1997, Kock and Bhat 1994, Simmons 2005, Srinivasulu and Srinivasulu 2012), respectively. Since the early 1990s, the Durga Das’s leaf-nosed bat has been reported only from the type locality, and in the absence of recent records the present status of this species remains unknown (Molur and Srinivasulu 2008). During recent surveys to document bat diversity in selected districts of Karnataka, we collected representatives of *Hipposideros* with no supplementary leaflets, from subterranean caves in Hanumanhalli and Therahalli villages, Kolar district, Karnataka. They were retained as voucher specimens, and after detailed analyses of the external, cranial and bacular morphology were identified as *H. durgadasi*. Here we provide the first record of *H. durgadasi* from southern India, and compare its morphology with that of other species in the *bicolor* group. As a result of the comparison process, we provide a description of the species, which is lacking in the current literature.

## Materials and methods

### Study Area

The general topography of the study area comprises rocky hills (900–1100 m of elevation) and low granitic hills, gently rolling expanses of sheet rocks interspersed with dry deciduous and scrub vegetation patches. The vegetation is dominated by tropical dry deciduous forests and tropical thorn forests (Champion and Seth 1968). Caves and cave systems were observed in the boulder hills, while subterranean caves were found predominantly in the low granitic hills.

### Surveys

Surveys were conducted in and around Kolar district (13°7′59.88″ N, 78°7′59.88″ E), Karnataka state, India, in November–December 2013 and May 2014. Because our goal was to locate roosting sites of bats in the area, we searched for crevices among boulders, subterranean caves and caverns, and old dilapidated temples and buildings. We surveyed four subterranean caves on a low hill at Hanumanhalli village, one of which was found to be actively harbouring bats. We conducted six mist-net night capture sections in this area. We also came across subterranean caves in the Therahalli village, located 5 km east of Hanumanhalli. At this site, and in the surrounding boulder hills, we conducted nine mist-net night capture sections.

### Morphological and morphometric analyses

A male and a female *H. durgadasi* were captured from both sites (totaling 4 individuals) and retained as voucher specimens. The individuals were photographed, prepared as fluid-preserved specimens, and deposited in the Natural History Museum of the Department of Zoology, Osmania University, Hyderabad, India (NHM.OU). External and craniodental measurements of the specimens were taken with a digital Vernier calliper accurate to 0.01mm. Specimens were identified following the criteria provided by Bates and Harrison (1997) and Srinivasulu et al. (2010). Their bacula were prepared following Topal (1958), measured with a oculometer, and identified based on Topal (1975). They were compared with representatives of *Hipposideros ater* Templeton, 1848, *Hipposideros cineraceus* Blyth, 1853, *Hipposideros fulvus* Gray, 1838, and *Hipposideros pomona* K. Andersen, 1918.

### Abbreviations used

**ZSIK**: Zoological Survey of India, Kolkata; **NHM.OU**: Natural History Museum, Osmania University, Hyderabad; ***External measurements***: **FA**: Forearm length; **HB**: Head Body length; **Tail**: Tail Length; **Ear**: Ear Length; **Hf**: Hindfoot length; **Hw**: Horseshoe width; **Tib**: Tibia length; **3mt**: Length of the third metacarpal; **4mt**: Length of the fourth metacarpal; **5mt**: Length of the fifth metacarpal; **1ph3mt**: First phalanx of the third metacarpal; **2ph3mt**: Second phalanx of the third metacarpal; **1ph4mt**: Length of the first phalanx of the fourth metacarpal; **2ph4mt**: Length of the second phalanx of the fourth metacarpal. ***Craniodental measurements***: **GTL**: Greatest length of skull; **CBL**: Condylobasal length; **CCL**: Condylocanine length; **ZB**: Zygomatic breadth; **BB**: Breadth of braincase; **CM^3^**: Maxillary toothrow length; **CM_3_**: Mandibular toothrow length; **M^3^-M^3^**: Posterior palatal width; **C^1^-C^1^**: Anterior palatal width; **M**: Mandibular length. ***Dentition***: **PM^2^**: First upper premolar; **PM^4^**: Second upper premolar; **M^1^**: First upper molar; **M^2^**: Second upper molar; **M^3^**: Third upper molar; **I_1_**: First lower incisor; **I_2_**: Second lower incisor; **PM_2_**: First lower premolar; **PM_4_**: Second lower premolar; **M_1_**: First lower molar; **M_2_**: Second lower molar; **M_3_**: Third lower molar.

## Taxon treatments

### Hipposideros durgadasi

Khajuria, 1970

#### Materials

- **Type status:** Holotype. **Occurrence:** catalogNumber: ZSIK Reg. No. V. 1906; occurrenceRemarks: Collected live; recordedBy: H. P. Agrawal; individualCount: 1; sex: Male; preparations: Whole animal (ETOH); skull extracted; **Taxon:** higherClassification: Animalia; Chordata; Vertebrata; Mammalia; Theria; Eutheria; Chiroptera; Hipposideridae; Hipposideros; durgadasi; kingdom: Animalia; phylum: Chordata; class: Mammalia; order: Chiroptera; family: Hipposideridae; genus: Hipposideros; specificEpithet: durgadasi; taxonRank: species; scientificNameAuthorship: Khajuria, 1970; vernacularName: Durga Das’s Leaf-nosed Bat; nomenclaturalCode: ICZN; taxonomicStatus: accepted; **Location:** country: India; stateProvince: Madhya Pradesh; verbatimLocality: Katangi Village; **Identification:** identificationID: Hipposideros durgadasi; identifiedBy: H. Khajuria; identificationReferences: Khajuria 1970; **Event:** eventDate: 1966-09-17; year: 1966; month: 09; day: 17; **Record Level:** type: PhysicalObject; language: en; rightsHolder: Zoological Survey of India, Government of India; bibliographicCitation: Khajuria H. 1970. A new leaf-nosed bat from central India. Mammalia, 34: 622–627.; institutionCode: ZSIK; basisOfRecord: PreservedSpecimen
- **Type status:** Paratype. **Occurrence:** catalogNumber: ZSIK Reg. No. V. 1928; occurrenceRemarks: Collected live; individualCount: 1; preparations: Whole animal (ETOH); skull extracted; **Taxon:** higherClassification: Animalia; Chordata; Vertebrata; Mammalia; Theria; Eutheria; Chiroptera; Hipposideridae; Hipposideros; durgadasi; kingdom: Animalia; phylum: Chordata; class: Mammalia; order: Chiroptera; family: Hipposideridae; genus: Hipposideros; specificEpithet: durgadasi; taxonRank: species; scientificNameAuthorship: Khajuria, 1970; vernacularName: Durga Das’s Leaf-nosed Bat; nomenclaturalCode: ICZN; taxonomicStatus: accepted; **Location:** country: India; stateProvince: Madhya Pradesh; verbatimLocality: Katangi Village; **Identification:** identificationID: Hipposideros durgadasi; identifiedBy: H. Khajuria; identificationReferences: Khajuria 1970; **Event:** eventDate: 1967-03-04; year: 1967; month: 03; day: 04; **Record Level:** type: PhysicalObject; language: en; rightsHolder: Zoological Survey of India, Government of India; bibliographicCitation: Khajuria H. 1970. A new leaf-nosed bat from central India. Mammalia, 34: 622–627.; institutionCode: ZSIK; basisOfRecord: PreservedSpecimen
- **Type status:** Other material. **Occurrence:** catalogNumber: NHM.OU.CHI.K10.2014; occurrenceRemarks: Collected live by mist netting; recordedBy: C. Srinivasulu; Aditya Srinivasulu; individualCount: 1; sex: Male; preparations: Whole animal (ETOH); skull extracted; **Taxon:** higherClassification: Animalia; Chordata; Vertebrata; Mammalia; Theria; Eutheria; Chiroptera; Hipposideridae; Hipposideros; durgadasi; kingdom: Animalia; phylum: Chordata; class: Mammalia; order: Chiroptera; family: Hipposideridae; genus: Hipposideros; specificEpithet: durgadasi; taxonRank: species; scientificNameAuthorship: Khajuria, 1970; vernacularName: Durga Das’s Leaf-nosed Bat; nomenclaturalCode: ICZN; taxonomicStatus: accepted; **Location:** country: India; stateProvince: Karnataka; verbatimLocality: Hanumanhalli, Kolar District; **Identification:** identificationID: Hipposideros durgadasi; identifiedBy: Chelmala Srinivasulu; Bhargavi Srinivasulu; Harpreet Kaur; **Event:** samplingProtocol: mist net; eventDate: 2014-05-12; year: 2014; month: 05; day: 12; **Record Level:** type: PhysicalObject; language: en; rightsHolder: Natural History Museum of Osmania University, Hyderabad; institutionCode: NHM.OU; basisOfRecord: PreservedSpecimen
- **Type status:** Other material. **Occurrence:** catalogNumber: NHM.OU.CHI.K40.2014; occurrenceRemarks: Collected live by mist netting; recordedBy: Bhargavi Srinivasulu; Tariq Ahmed Shah; individualCount: 1; sex: Female; preparations: Whole animal (ETOH); skull extracted; **Taxon:** higherClassification: Animalia; Chordata; Vertebrata; Mammalia; Theria; Eutheria; Chiroptera; Hipposideridae; Hipposideros; durgadasi; kingdom: Animalia; phylum: Chordata; class: Mammalia; order: Chiroptera; family: Hipposideridae; genus: Hipposideros; specificEpithet: durgadasi; taxonRank: species; scientificNameAuthorship: Khajuria, 1970; vernacularName: Durga Das’s Leaf-nosed Bat; nomenclaturalCode: ICZN; taxonomicStatus: accepted; **Location:** country: India; stateProvince: Karnataka; verbatimLocality: Hanumanhalli, Kolar District; **Identification:** identificationID: Hipposideros durgadasi; identifiedBy: Chelmala Srinivasulu; Bhargavi Srinivasulu; Harpreet Kaur; **Event:** samplingProtocol: mist net; eventDate: 2014-05-12; year: 2014; month: 05; day: 12; **Record Level:** type: PhysicalObject; language: en; rightsHolder: Natural History Museum of Osmania University, Hyderabad; institutionCode: NHM.OU; basisOfRecord: PreservedSpecimen
- **Type status:** Other material. **Occurrence:** catalogNumber: NHM.OU.CHI.K46.2014; occurrenceRemarks: Collected live by mist netting; recordedBy: C. Srinivasulu; Harpreet Kaur; individualCount: 1; sex: Female; preparations: Whole animal (ETOH); skull extracted; **Taxon:** higherClassification: Animalia; Chordata; Vertebrata; Mammalia; Theria; Eutheria; Chiroptera; Hipposideridae; Hipposideros; durgadasi; kingdom: Animalia; phylum: Chordata; class: Mammalia; order: Chiroptera; family: Hipposideridae; genus: Hipposideros; specificEpithet: durgadasi; taxonRank: species; scientificNameAuthorship: Khajuria, 1970; vernacularName: Durga Das’s Leaf-nosed Bat; nomenclaturalCode: ICZN; taxonomicStatus: accepted; **Location:** country: India; stateProvince: Karnataka; verbatimLocality: Therahalli, Kolar District; **Identification:** identificationID: Hipposideros durgadasi; identifiedBy: Chelmala Srinivasulu; Bhargavi Srinivasulu; Harpreet Kaur; **Event:** samplingProtocol: mist net; eventDate: 2014-05-13; year: 2014; month: 05; day: 13; **Record Level:** type: PhysicalObject; language: en; rightsHolder: Natural History Museum of Osmania University, Hyderabad; institutionCode: NHM.OU; basisOfRecord: PreservedSpecimen
- **Type status:** Other material. **Occurrence:** catalogNumber: NHM.OU.CHI.K48.2014; occurrenceRemarks: Collected live by mist netting; recordedBy: Aditya Srinivasulu; C. Srinivasulu; individualCount: 1; sex: Male; preparations: Whole animal (ETOH); skull extracted; **Taxon:** higherClassification: Animalia; Chordata; Vertebrata; Mammalia; Theria; Eutheria; Chiroptera; Hipposideridae; Hipposideros; durgadasi; kingdom: Animalia; phylum: Chordata; class: Mammalia; order: Chiroptera; family: Hipposideridae; genus: Hipposideros; specificEpithet: durgadasi; taxonRank: species; scientificNameAuthorship: Khajuria, 1970; vernacularName: Durga Das’s Leaf-nosed Bat; nomenclaturalCode: ICZN; taxonomicStatus: accepted; **Location:** country: India; stateProvince: Karnataka; verbatimLocality: Therahalli, Kolar District; **Identification:** identificationID: Hipposideros durgadasi; identifiedBy: Chelmala Srinivasulu; Bhargavi Srinivasulu; Harpreet Kaur; **Event:** samplingProtocol: mist net; eventDate: 2014-05-13; year: 2014; month: 05; day: 13; **Record Level:** type: PhysicalObject; language: en; rightsHolder: Natural History Museum of Osmania University, Hyderabad; institutionCode: NHM.OU; basisOfRecord: PreservedSpecimen

#### Description

Because no detailed description of the Durga Das’s leaf-nosed bat, *Hipposideros durgadasi*, is available, we provide one here based on the recent collections.

##### External Characters (Fig. 1, Table 1)

**Figure 1.**
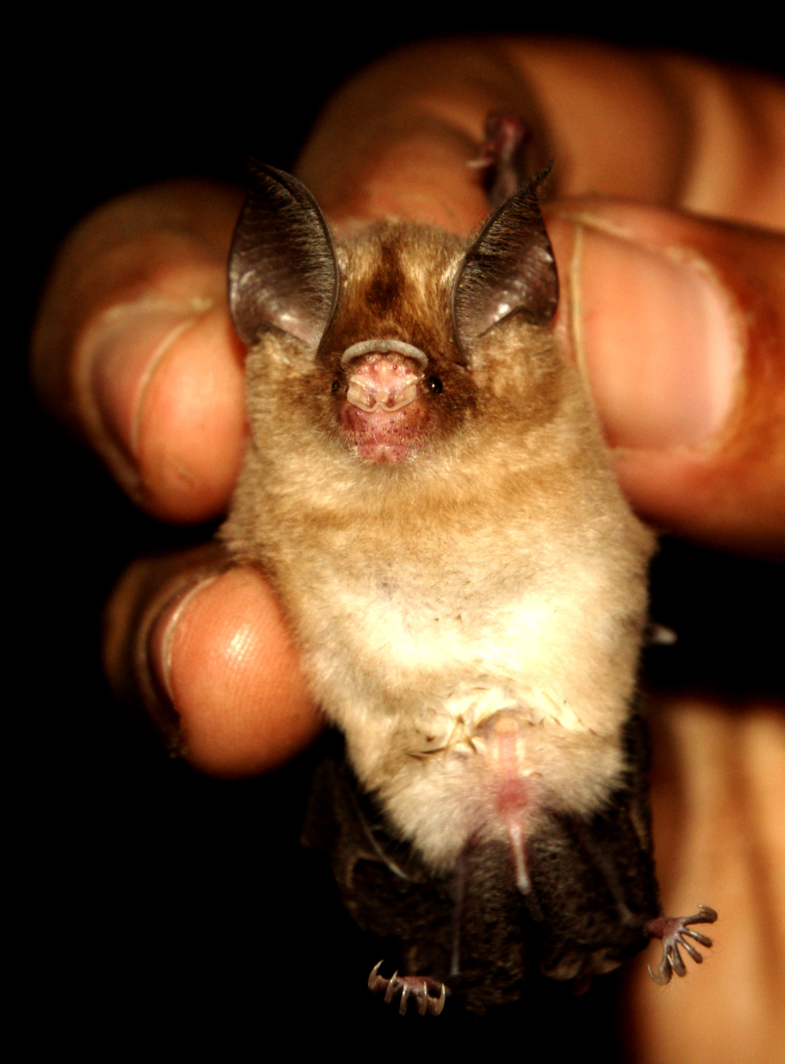
Durga Das’s leaf-nosed bat, *Hipposideros durgadasi*, male (NHM.OU.CHI. K10.2014) from Hanumanhalli, Kolar district, Karnataka, India

**Figure 2.**
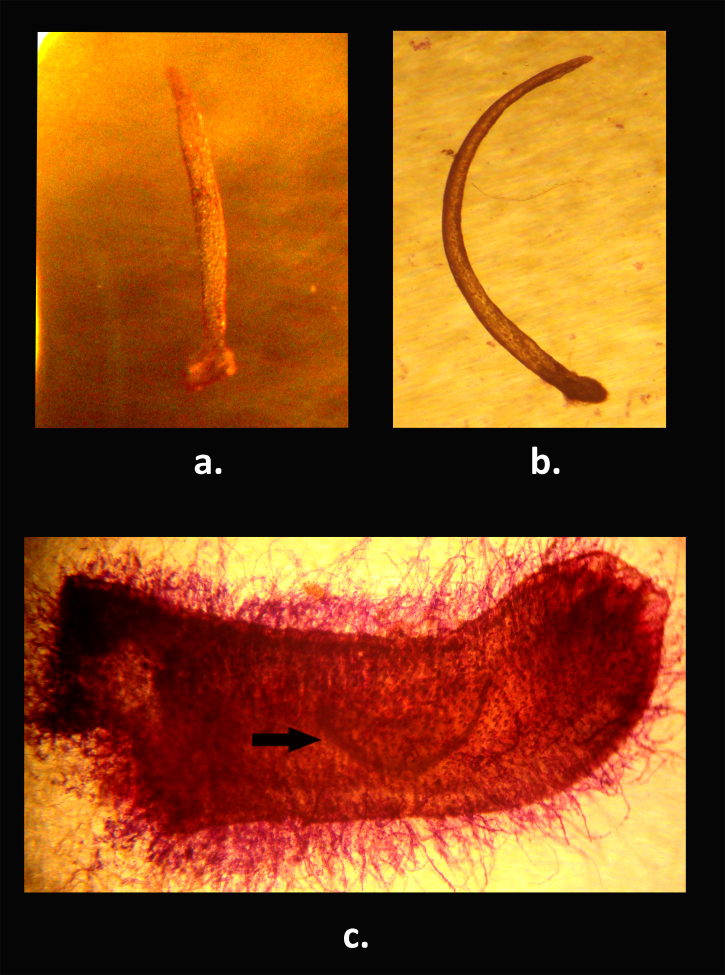
Baculum of the Durga Das’s leaf-nosed bat, *Hipposideros durgadasi*, (NHM.OU.CHI. K10.2014). A. Dorsal view; B. lateral view; C. positioning of the baculum (indicated by an arrow) in the penis of *H. durgadasi*

A small species of *Hipposideros* (Fig. 1), with forearm length ranging between 34.45–35.95mm (Table 1). Ears small (12.70–13.48mm Table 1), with a well-defined antitragus, bluntly rounded tip, and ten ridges, of which all, except the top 2, show bifurcation toward the outer edge of the pinna. A small and simple noseleaf present on the muzzle, with the greatest width ranging between 3.86–4.25mm; no supplementary leaflets. The anterior leaf has a median emargination and is covered throughout with short, stiff black hairs. The internarial septum is well developed, with a short base and a bulbous apex. Nostrils are oval in shape and possess narial lappets on the outer margin. A pair of vibrissae is present at the broadest portion of the anterior leaf. The intermediate leaf has two pairs of vibrissae, one pair on each side; it is smaller in size than the anterior and posterior leaves. Upper border of the intermediate leaf broadly convex, covered with long, stiff black hairs and with a slight projection toward the middle. The posterior leaf has three ill-defined septa dividing it into four cells. The posterior leaf has an evenly rounded convex anterior border. Two pairs of vibrissae situated behind the posterior leaf, on the lateral aspect. An ill-defined frontal sac located in mid-line behind the posterior leaf is barely visible in male specimens; not clearly defined in female specimens. Feet are small. Fourth metacarpal exceeds the third and the fifth in length, the fifth being the shortest. The combined length of the phalanges of the third metacarpal slightly exceeds the length of the metacarpal. The first phalanx of the fourth metacarpal slightly exceeds the second in length. The tail is long (21.21–22.94mm) and enclosed in the interfemoral membrane, except the extreme tip, which ranges from 1.22 to 2.38mm. Wing and interfemoral membranes hairless, dark above and below, and attached to the tibia. However, the point of attachment of the interfemoral membrane is located above that of the wing membrane. In both sexes, the basal half of the dorsal hairs are cream in colour and the other half is pale brown. The dorsal fur is interspersed with long black hairs. Ventral fur is cream with only the apices being pale brown. The penis is cylindrical, long and has a curved tip. The baculum (length [from the base to the tip]= 1.47–1.49mm) is lodged in the middle of the penis shaft and has a characteristic semicircular shape. This structure is also slightly wider at the base, where the dorsal side is concave, presents a constriction just beyond the base, and ends with a pointed tip (Fig. 2).

##### Cranial characters (Fig. 3)

**Figure 3.**
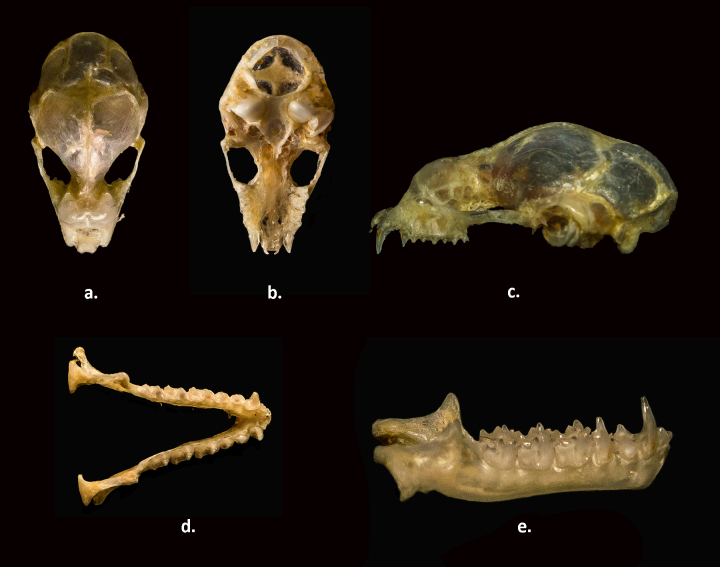
Skull and mandible of Durga Das’s leaf-nosed bat *Hipposideros durgadasi* (NHM.OU.CHI. K10.2014). a. maxilla dorsal view; b. maxilla ventral view; c. maxilla lateral view; d. mandible dorsal view; e. mandible lateral view

The skull is small (Fig. 3), with an average condylocanine length of 12.68mm (12.51–12.97mm) (Table 3). Three pairs of nasal inflations present on the rostrum, the anterior pair duct-like, curved; the posterior pair small and spherical; the lateral pair is the largest of the three and spherical in shape. The rostrum is low, flat and seems horizontal. The sagittal crest is prominent in the frontal region and inconspicuous posteriorly. The zygomata are slender, with no dorsal process, flared postero-laterally, and a little wider than the braincase. There is a slight depression at the fronto-parietal suture. The anterior border of the mesopterygoid space is U–shaped. The cochlea exceeds in size the tympanic bulla. The coronoid process of each half mandible exceeds the height of the canine. The angular process is well developed and angled outward.

##### Dentition (Fig. 3)

Upper toothrow (C-M^3^) averages 4.74mm (4.67–4.78mm) (Table 3). The upper incisors are small, tricuspidate and wedge-shaped (Fig. 3). The upper canine is robust, with a well-developed cingulum. The first upper premolar (PM^2^) is minute, aligned in the toothrow, and wedged between the canine and the comparatively large second upper premolar (PM^4^), giving rise to a gap between the two teeth. M^1^ and M^2^ have the typical W–shaped cusp pattern, with well-developed parastyle, mesostyle, and metastyle, and four commissures. In M^1^ and M^2^ the paracone exceeds the size of the metacone. The crown area of M^1^ is equal to that of M^2^. The M^3^ is two-thirds the crown area of M^2^. the parastyle in M^3^ is well-developed, the mesostyle is comparatively less developed, and the metastyle is absent. The paracone in M^3^ is well-developed, and the mesocone is very short in comparison with the paracone. The metacone is much reduced in size in comparison with the paracone. Three commissures are present, of which two are well-developed and the third is very short. Two pairs of tricuspidate lower incisors are present, the second (I_2_) is situated partially behind the first (I_1_). The first lower premolar (PM_2_) is half to two thirds the height of the second lower premolar (PM_4_). The protoconid is the dominant cusp in all the lower premolars. The M_3_ is smaller than the M_1_ and M_2_ in crown area and height.

## Discussion

The average forearm length of *Hipposideros durgadasi* is shorter than that of *H. ater*, *H. fulvus*, and *H. pomona* (35.4mm vs 36.3 mm, 40.4mm, and 39.0mm, respectively [Table 2]), and it exceeds the forearm length of *H. cineraceus* (35.4mm *H. durgadasi* vs. 34.7mm *H. cineraceus*) (Table 2). Ears are also considerably shorter than those of *H. ater*, *H. cineraceus*, *H. fulvus*, and *H. pomona* (13.02mm vs. 17.6mm, 15.2mm, 22.0mm, 18.8mm, respectively). The fourth metacarpal exceeds the length of the third and the fifth metacarpals, and the fifth metacarpal is the shortest. In *H. ater*, *H. fulvus* and *H. pomona* the third metacarpal is the shortest and the fourth metacarpal is the longest. The condylocanine length averages shorter than in *H. ater*, *H. cineraceus*, *H. fulvus*, and *H. pomona* (12.7mm vs. 13.6mm, 13.2mm, 15.6mm, 15.5mm, respectively) (Table 3). The palate is shorter in comparison to *H. ater*, *H. cineraceus*, *H. fulvus*, and *H. pomona* (Table 3). The baculum of *H. durgadasi* is strongly bowed and semi-circular in shape, while the baculum of *H. ater* is straight with a bluntly rounded tip; that of *H. cineraceus* is straight with a bifid tip; and the baculum of *H. fulvus*, although resembling that of *H. ater*, has a slightly curved shaft (Topal 1975).

Studies by Khajuria (Khajuria 1970) on specimens of *H. durgadasi* collected in Jabalpur, Madhya Pradesh, India, showed that the tail vertebrae projected 2.5–4.0mm beyond the interfemoral membrane, which was not the case in the specimens in the collection of Bombay Natural History Society (3 specimens including 1 adult male, 1 adult female, and 1 subadult male) assigned to *Hipposideros cineraceus cineraceus* from Salem district, Tamil Nadu. These specimens present a much shorter projection of tail vertebrae from beyond the interfemoral membrane. During the present study, from we observed that the tail tip of the specimens Karnataka projected between 1.3–2.4mm beyond the interfemoral membrane. *Hipposideros durgadasi* was first described by Khajuria in 1970 as a subspecies of *Hipposideros cineraceus*, but differed with respect to skull and external measurements (forearm longer and ears shorter than in *H. cineraceus*). Based on the strongly bowed semi-circular baculum, Topal (1975) elevated *durgadasi* to the species level. Khajuria (1982) confirmed the observations of Topal (1975). The voucher specimens assigned to *H. cineraceus* collected from south India, including Tamil Nadu and Karnataka (Bhat and Jacob 1990, Sreepada et al. 1993), were provisionally included under *Hipposideros ater* by Bates and Harrison (1997), who considered that *H. cineraceus* was restricted in distribution to the Himalayan belt. For other authors, however, this species was distributed throughout India (Blanford 1881, Wroughton 1918, Khajuria 1970). Furthermore, Khajuria (1970) suggested that the *H. cineraceus* from south India may in fact be *Hipposideros durgadasi*.

**Figure 4.**
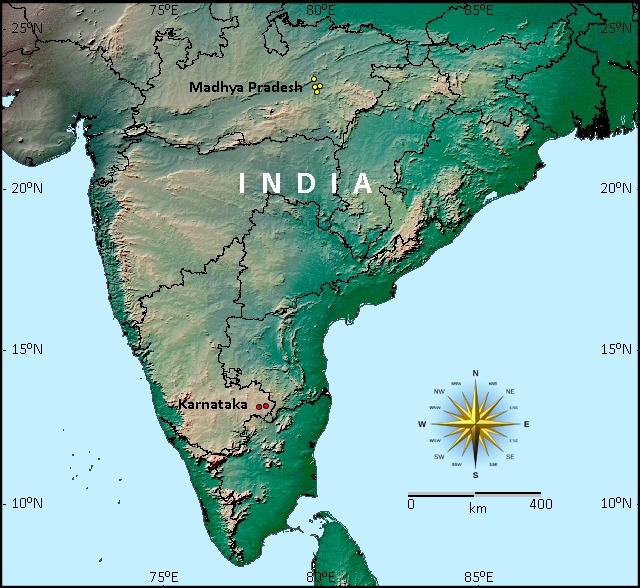
Distribution map of Durga Das's leaf-nosed bat, *Hipposideros durgadasi*, showing known locality records (in yellow) from Madhya Pradesh and new records (in red) in Karnataka, India.

Our record of *Hipposideros durgadasi* from Kolar district, Karnataka, constitutes the first record of this species from South India (Fig. 4) supported by voucher specimens. The other known localities are the Richchai, Katanga, Katangi, Gwarighat villages of Jabalpur district, Madhya Pradesh in Central India (Khajuria 1970, Khajuria 1980, Topal 1975). With the new locality record, the known distribution range of *H. durgadasi* is extended by more than 1300 km southward from the known localities in central India. In Hanumanhalli village, *H. durgadasi* was found roosting in narrow subterranean caves and sharing roost with *H. fulvus*, *H. hypophyllus*, *H. speoris*, and *Rhinopoma hardwickii*. In Therahalli village, the subterranean cave where we collected bats was harbouring only populations of *H. durgadasi*. Our results confirm the initial observations of Khajuria (1970), reinforcing the importance of further studies on *H. cineraceus*, *H. ater* and *ater-* like taxa to clarify the distribution of *H. durgadasi* in other parts of south India. More extensive surveys might also help reveal the actual distribution range of this endemic species.

### Threats and Conservation

The subterranean caves where *H. durgadasi* occurs, especially in Hanumanhalli village, Kolar, Karnataka, are facing great threat due to illegal granite mining. This activity is presently progressing to within a few hundred feet from the roost of this species, endangering its population and those from congeners that share this roost. Two additional roosting sites in subterranean caves at the same site were abandoned two years ago due to human-lit fires for the purpose of cutting the slabs of granite (information provided by locals). There exists an urgent need to curb this illegal mining activity to protect population of *H. durgadasi* and its congeners, including the Kolar leaf-nosed bat, *H. hypophyllus*, another endemic and endangered cave dwelling species.

## Supplementary Material

XML Treatment for Hipposideros durgadasi

## Figures and Tables

**Table 1.** External and craniodental measurements (in mm) of the vouchers of *Hipposideros durgadasi* from Kolar, Karnataka, India (see Material and Methods for abbreviations).

	**NHM.OU.CHI.K10.2014**	**NHM.OU.CHI.K40.2014**	**NHM.OU.CHI.K46.2014**	**NHM.OU.CHI.K48.2014**
**External measurements**				
**FA**	35.43	35.79	35.95	34.45
**HB**	41.12	37.60	37.40	36.45
**Tail**	22.94	22.11	21.21	21.57
**Ear**	12.70	13.21	13.48	12.70
**HF**	5.66	6.70	5.55	5.10
**TIB**	16.07	16.19	16.43	15.38
**3mt**	27.68	28.00	27.80	26.12
**4mt**	29.13	29.61	29.55	27.62
**5mt**	27.30	27.71	27.05	25.75
**1ph3mt**	15.11	15.00	14.67	13.78
**2ph3mt**	15.22	15.47	15.09	14.0
**1ph4mt**	8.24	8.76	8.35	8.27
**2ph4mt**	8.09	7.90	8.26	7.63
**Hw**	4.15	4.25	4.12	3.86
**Tail Tip**	1.22	2.38	1.28	2.14
**Skull measurements**				
**GTL**	15.42	14.90	14.97	14.82
**CBL**	13.25	13.20	12.80	12.94
**CCL**	12.97	12.68	12.51	12.59
**ZB**	6.98	7.30	7.32	7.40
**BB**	7.97	7.61	7.83	7.95
**C-M^3^**	4.76	4.78	4.76	4.67
**C^1^-C^1^**	2.87	2.89	2.83	2.84
**M^3^-M^3^**	5.07	4.93	4.75	4.78
**M**	8.71	8.76	8.35	8.34
**C-M_3_**	5.03	5.10	5.09	4.57

**Table 2.** Comparison of external measurements (in mm) of the specimens of *Hipposideros durgadasi* from Karnataka(present study [*n* = 4]) with those from the literature for *Hipposideros durgadasi* (from Japalpur; measurements for the holotype [male] and other 23 specimens), *Hipposideros ater* (from India and Sri Lanka), *Hipposideros cineraceus* (from India), *Hipposideros fulvus* (from India) and *Hipposideros pomona* (from southern India). Measurements for *H. durgadasi* from Japalpur were obtained from Khajuria (1970), and those for *H. ater*, *H. fulvus* and *H. pomona* were obtained from Bates and Harrison (1997). See Material and Methods for abbreviations.

**Species**	**FA**	**HB**	**Tail**	**Ear**	**Hf**	**Tib**	**3mt**	**4mt**	**5mt**	**1ph3mt**	**2ph3mt**	**1ph4mt**	**2ph4mt**
***Hipposideros durgadasi* Karnataka** **n=4 (2♂♂, 2♀♀)**	35.4± 0.67 34.45-35.95	38.14± 2.04 36.45-41.12	21.95± 0.75 21.21-22.94	13.02± 0.38 12.70-13.48	5.75± 0.67 5.1-6.7	16.01± 0.45 15.38-16.43	27.40± 0.86 26.12-28.0	28.97± 0.92 27.62-29.61	26.95± 0.84 25.75-27.71	14.64± 0.60 13.78-15.11	14.94± 0.64 14.0-15.47	8.405± 0.24 8.24-8.76	7.97± 0.27 7.63-8.26
***Hipposideros durgadasi* (Jabalpur; Holotype [ZSIK Reg. No. V. 1906])**	37.5	-	24.5	15.0	7.0	17.0	27.0	29.0	25.5	15.0	-	8.5	-
***Hipposideros durgadasi* (Jabalpur; n=23 [12♂♂, 11♀♀])**	37.0 36.0-37.50	-	23.5 21.50-29.0	15.4 13.0-19.0	6.5 5.5-8.0	-	-	-	-	-	-	-	-
***Hipposideros ater***	36.3± 0.93 4.9-38.0	42.3± 2.8 38.0-48.0	24.7± 2.1 20.0-30.0	17.6± 1.4 14.8-20.0	6.7± 0.5 5.3-7.2	16.3± 0.6 15.2-17.8	27.5± 1.0 26.1-30.1	29.2± 1.0 27.2-32.2	28.0± 1.1 26.2-31.2	15.6± 0.7 14.3-17.5	15.8± 0.8 14.3-17.4	9.8± 0.6 8.7-10.9	8.3± 0.6 7.0-9.2
***Hipposideros cineraceus***	34.7± 0.9 33.0-36.3	37.1± 2.7 33.0-42.0	26.1± 2.1 22.0-30.0	15.2± 1.2 13.0-17.0	6.5± 0.5 6.0-7.0	15.3± 0.8 13.8-16.7	25.8± 0.9 24.4-26.6	27.7± 0.6 26.9-28.8	27.0± 0.5 26.2-27.8	15.4± 0.6 14.3-16.2	13.4± 0.7 12.5-15.3	9.3± 0.6 8.4-11.2	7.0± 0.8 6.2-8.6
***Hipposideros fulvus***	40.4± 1.5 38.4-44.0	47.0± 3.0 40.0-50.0	29.5± 3.0 20.0-35.0	22.0± 1.3 19.0-26.0	7.6± 1.3 6.0-9.8	18.5± 1.0 16.5-20.7	29.2± 0.9 27.3-31.2	31.2± 1.1 28.3-33.9	30.8± 1.5 28.7-33.1	17.5± 0.8 16.1-18.9	18.0± 0.8 16.2-19.5	11.0± 0.5 10.0-12.0	9.6± 0.7 8.2-11.2
***Hipposideros pomona***	39.0± 0.7 38.1-39.7	-	-	18.8± 0.3 18.5-19.0	-	-	29.1± 0.5 28.6-29.5	31.0± 0.6 30.3-31.5	30.6± 0.3 30.3-30.9	-	-	-	-

**Table 3.** Comparison of cranio-dental measurements (in mm) of *Hipposideros durgadasi* from Kolar District, Karnataka (present study) and literature measurements of *Hipposideros durgadasi*, *Hipposideros ater*, *Hipposideros cineraceus*, *Hipposideros fulvus* and *Hipposideros pomona*

**Craniodental measurements**	**GTL**	**CBL**	**CCL**	**ZB**	**BB**	**C-M^3^**	**C^1^-C^1^**	**M^3^-M^3^**	**M**	**C-M_3_**
***Hipposideros durgadasi* Karnataka** **n=4 (2♂♂, 2♀♀)**	15.02± 0.26 14.82-15.42	13.04± 0.21 12.8-13.25	12.68± 0.20 12.5-12.97	7.25± 0.18 6.98-7.40	7.84± 0.16 7.61-7.97	4.74± 0.04 4.67-4.78	2.85± 0.02 2.83-2.89	4.88± 0.14 4.75-5.07	8.54± 0.22 8.34-8.76	4.94± 0.25 4.57-5.1
***Hipposideros durgadasi* Jabalpur Holotype (ZSIK Reg. No. V. 1906) (Khajuria, 1970)**	14.7	13.0	-	8.1	7.7	5.0	4.0	5.5	9.2	5.3
***Hipposideros durgadasi* Jabalpur** **n=15 (11♂♂, 4♀♀) (Khajuria, 1970)**	15.0 14.5-16.1	13.1 13.0-13.9	-	7.1 6.9-9.0	7.4 7.0-8.5	5.2 5.0-6.0	3.2 3.0-3.7	5.5 5.0-5.8	9.2 9.0-9.5	5.3 5.0-6.0
***Hipposideros ater*** **India, Sri Lanka** **(Bates and Harrison, 1997)**	16.0± 0.3 15.4-16.7	-	13.6± 0.3 13.2-14.2	8.1± 0.1 7.7-8.3	8.1± 0.2 7.5-8.5	5.3± 0.2 5.1-5.7	3.6± 0.1 3.3-3.8	5.5± 0.1 5.1-5.8	9.8± 0.3 9.4-10.2	5.7± 0.2 5.2-6.1
***Hipposideros cineraceus* India** **(Bates and Harrison, 1997)**	15.6± 0.3 15.2-16.2	-	13.2± 0.3 12.7-13.7	7.3± 0.2 6.9-7.6	7.8± 0.2 7.2-8.2	5.0± 0.3 4.9-5.3	3.0± 0.1 2.7-3.1	4.9± 0.1 4.6-5.1	9.0± 0.2 8.8-9.4	5.4± 0.2 5.2-5.8
***Hipposideros fulvus*** **India** **(Bates and Harrison, 1997)**	18.0± 0.3 17.2-18.6	-	15.6± 0.3 15.0-16.4	9.2± 0.2 8.6-9.6	8.3± 0.5 7.5-9.4	6.3± 0.2 6.0-6.9	4.0± 0.2 3.6-4.4	6.3± 0.2 5.8-6.8	11.5± 0.3 11.1-12.0	6.8± 0.2 6.4-7.5
***Hipposideros pomona*** **S. India** **(Bates and Harrison, 1997)**	16.6± 0.1 16.5-16.7	-	14.4± 0.2 14.2-14.6	8.2± 0.3 7.9-8.4	-	5.6± 0.1 5.5-5.7	3.4± 0.0 3.4-3.4	5.4± 0.1 5.3-5.5	9.9± 0.2 9.8-10.1	6.0± 0.0 6.0-6.0
